# Association of the protective effect of telmisartan on hearing loss among patients with hypertension

**DOI:** 10.3389/fneur.2024.1410389

**Published:** 2024-08-27

**Authors:** Jung-Joon Cha, Yunjin Yum, Yong Hyun Kim, Eung Ju Kim, Yoon Chan Rah, Euyhyun Park, Gi Jung Im, Jae-Jun Song, Sung-Won Chae, June Choi, Hyung Joon Joo

**Affiliations:** ^1^Division of Cardiology, Department of Internal Medicine, Korea University Anam Hospital, Seoul, Republic of Korea; ^2^Department of Biostatistics, Korea University College of Medicine, Seoul, Republic of Korea; ^3^Division of Cardiology, Department of Internal Medicine, Korea University Ansan Hospital, Ansan, Republic of Korea; ^4^Division of Cardiology, Department of Internal Medicine, Korea University Guro Hospital, Seoul, Republic of Korea; ^5^Department of Otorhinolaryngology-Head and Neck Surgery, Korea University Ansan Hospital, College of Medicine, Korea University, Ansan, Republic of Korea; ^6^Department of Otorhinolaryngology-Head and Neck Surgery, Korea University Anam Hospital, College of Medicine, Korea University, Seoul, Republic of Korea; ^7^Department of Otorhinolaryngology-Head and Neck Surgery, Korea University Guro Hospital, College of Medicine, Korea University, Seoul, Republic of Korea; ^8^Department of Medical Informatics, Korea University College of Medicine, Seoul, Republic of Korea; ^9^Korea University Research Institute for Medical Bigdata Science, College of Medicine, Korea University, Seoul, Republic of Korea

**Keywords:** telmisartan, hearing loss, angiotensin II receptor blocker, PPAR γ agonist, hypertension

## Abstract

**Aim:**

Hearing loss, affecting a significant portion of the global population, is prevented with peroxisome proliferator-activated receptor γ agonism. Understanding potential protective treatments is crucial for public health. We examine the effect of telmisartan, an antihypertensive drug and partial peroxisome proliferator-activated receptor γ agonist, on hearing loss in patients with hypertension.

**Method and results:**

This retrospective cohort analysis used data from the OMOP Common Data Model database, encompassing information from three tertiary institutions in South Korea. The study included a substantial sample size of 860,103 people diagnosed with hypertension. The study included individuals who had been medically diagnosed with hypertension and had been prescribed antihypertensive drugs, including telmisartan. The study design was established to evaluate the comparative effects of telmisartan and other hypertension medications on hearing loss. We used propensity score matching (PSM) to create a balanced cohort, reducing potential biases between the telmisartan and non-telmisartan groups. From the initial 860,103 patients with hypertension, a propensity score matched cohort was derived from 20,010 patients, with 2,193 in the telmisartan group. After PSM, lower incidence of total hearing loss was observed in the telmisartan group compared to the non-telmisartan group during the 3-year follow-up (0.5% vs. 1.5%, log-rank *p* = 0.005). In subgroup analysis, this study showed consistent results that lower incidence of total hearing loss was higher in the telmisartan group than in the non-telmisartan group.

**Conclusion:**

Telmisartan was associated with reducing certain types of hearing loss in patients with hypertension. Further research is needed to confirm these findings and understand the mechanisms.

## 1 Introduction

The World Health Organization (WHO) estimates that as of 2021, nearly 5% of the global population suffers from disabling hearing loss (1, 2). In South Korea, the Korea National Health and Nutrition Examination Survey indicated that the prevalence of bilateral hearing loss in adults aged 20 and over was approximately 13.3% in 2012 (3). Hearing loss can impact an individual's life, leading to challenges in communication, emotional health, and cognitive decline (4). Common causes of hearing loss include presbycusis (age-related hearing loss, particularly in those over 65), noise exposure (both occupational and recreational), otosclerosis, and Meniere's disease (5). Heart disease, diabetes, and the use of ototoxic drugs are also identified as risk factors for hearing loss (5).

Telmisartan, a widely used antihypertensive medication, also functions as a partial peroxisome proliferator-activated receptor γ (PPAR γ) agonist (6, 7). Research has suggested that PPAR γ agonists may protect against hearing loss (8). Studies involving PPAR γ agonists, such as thiazolidinediones, have reduced ototoxicity and noise-induced hearing loss (9). *In vitro* studies have further confirmed that telmisartan can mitigate hearing loss associated with ototoxic drugs (10). However, there is no clinical evidence to establish whether telmisartan effectively prevents hearing loss in patients.

The current study aimed to evaluate the preventive effect of telmisartan on hearing loss, comparing it with non-telmisartan treatments based on data from a multicenter retrospective registry.

## 2 Methods

### 2.1 Source of data

This was a pooled, retrospective, observational cohort study. The Observational Medical Outcomes Partnership (OMOP) Common Data Model (CDM) database of three tertiary institutions in Korea (Korea University Anam Hospital, Korea University Guro Hospital, and Korea University Ansan Hospital) was used for data collection. The Observational Health Data Sciences and Informatics partnership provides a data schema standardizing hospital electronic health record (EHR) in the OMOP-CDM database. The OMOP-CDM database contains complete information on healthcare services, including demographics, diagnoses, prescriptions, medical equipment, and procedure records. All prescribed medicines were recorded and categorized according to chemical composition and dosage. Individual diagnoses were coded using the International Classification of Diseases, 10th Revision (ICD-10). The OMOP-CDM database assigned a concept identifier (ID) that correlated to an ICD-10 code. The OMOP-CDM database was directly queried using Microsoft's structured query language. The institutional review board of the Korea University Medical Center (Seoul, Korea) approved the study methodology. It waived the requirement for informed consent because the OMOP-CDM database contained de-identified, anonymous data.

### 2.2 Study population and design

A total of 860,103 patients who sought outpatient treatment for hypertension at three tertiary hospitals between January 2017 and June 2021 were screened. Patients who were ≥18 years of age and had been initiated and maintained with at least two antihypertensive drugs as combination therapy were selected from the OMOP-CDM database of three hospitals. Antihypertensive drugs included angiotensin converting enzyme inhibitors, angiotensin II receptor blockers (ARBs), beta-blockers, dihydropyridine-calcium channel blockers (DHP-CCB), non-dihydropyridine calcium channel blockers, and diuretics. The index day was the 1^st^ day on which at least two antihypertensive medications were prescribed as combination therapy. Patients prescribed antihypertensives for < 30 days within the first 60 days of the index day were excluded. Patients were excluded if they were taking two or more ARBs, were diagnosed with hearing loss, Meniere's disease, end-stage renal disease on dialysis, heart failure, myocardial infarction, and stroke within 1 month of the index day. Overall, 2,193 patients were prescribed combination therapy with telmisartan (telmisartan group), and 17,817 patients were prescribed another ARB users (non-telmisartan group). To reduce the effect of selection bias, we conducted a propensity score matching (PSM) analysis for the comparison between the two groups.

### 2.3 Study variables and outcomes

Data on patient demographics, medical histories, laboratory test results, medications, and BPs were collected. Diabetes mellitus was defined as a fasting plasma glucose level ≥126 mg/dL, hemoglobin A1c level ≥6.5%, antidiabetic drug use, or OMOP-CDM concept ID for diabetes mellitus. Dyslipidemia was defined as serum total cholesterol ≥200 mg/dL, low-density lipoprotein cholesterol ≥130 mg/dL, triglycerides ≥150 mg/dL, high-density lipoprotein cholesterol < 40 mg/dL, taking statins or ezetimibe, or OMOP-CDM concept ID for dyslipidemia. Chronic kidney disease was defined as an estimated glomerular filtration rate of < 60 mL/min/1.73 m^2^ or proteinuria ≥1+ on routine urine analysis. Microalbuminuria was defined as a urinary albumin-to-creatinine ratio of more than 35 in women or more than 25 in men.

From 2017 to 2022, the dates and causes of death were extracted from death certificates in the EHR. Clinical events were determined using the ICD codes for hearing loss. The primary outcome was the incidence of any hearing loss, defined as the composite of bilateral hearing loss and unilateral hearing loss from 30 days to 3 years from the index day. The grades of hearing loss were categorized according to the WHO grades. Briefly, the corresponding audiometric ISO values of mild, moderate, and severe or deaf were 26–40 dB, 41–60 dB, and 61 or greater, respectively. Negative control outcomes were assessed using clinical diagnostic codes.

### 2.4 Statistical analysis

Categorical variables were reported as numeric values (percentages), while continuous variables were reported as means ± standard deviation. Categorical variables were compared using the χ^2^ test or Fisher's exact test. Continuous variables were compared using a parametric unpaired *t*-test or non-parametric Mann–Whitney test between the two groups. The likelihood of receiving telmisartan was quantified for PSM analysis using a multivariable logistic regression model. Telmisartan was used as the dependent variable, and all previously specified baseline characteristics were included in the model. After computing the expected probabilities, we matched each patient in the telmisartan initiators with those in the active comparators at a 1:3 ratio using the nearest neighbor method, with a caliper width equal to 0.1 of the standard deviation of the logit propensity score. Using the standardized mean difference (SMD), the balance of baseline features between telmisartan users and active comparators was examined; a SMD of < 0.15 indicated a minimal difference.

The cumulative rates of the study outcomes were computed using the Kaplan–Meier analyses, and *p* values were calculated using the log-rank test. The risks of the study outcomes were evaluated using a Cox proportional hazard model and reported as hazard ratios (HRs) and 95% confidence intervals (CIs). R Statistical Software (version 4.1.2; R Foundation for Statistical Computing, Vienna, Austria) was used to conduct statistical analyses, and a *p-*value < 0.05 was considered statistically significant.

## 3 Results

A total of 20,010 patients were selected for the final analysis. Of these, 2,193 (11.0%) patients were prescribed combination therapy with telmisartan (telmisartan group). After 1:3 matching, 6,012 patients were included in the PSM cohort (telmisartan group, *n* = 1,503; non-telmisartan group, *n* = 4,509).

### 3.1 Clinical characteristics of the study population

The baseline characteristics of the patients according to the use of telmisartan before and after PSM are summarized in Table 1. Before PSM, the average age, proportion of women, smoking prevalence, chronic kidney disease, and malignancy rate were lower in the telmisartan group compared to the non-telmisartan group. In addition, the telmisartan group had a lower usage of beta-blockers, diuretics, ototoxic drugs, and aspirin compared to the non-telmisartan group, with all differences statistically significant (*p* < 0.001). Blood pressure, total cholesterol, triglyceride, glucose, and high sensitivity C reactive protein levels were not statistically significant between the groups. The baseline characteristics after PSM reveal no significant differences in demographic and clinical parameters. In addition, demographics, comorbidities, medications, and laboratory values at baseline were well balanced to an SMD of < 0.15.

**Table 1:** Demographic and clinical characteristics before and after propensity score matching.

	**Propensity score-matched cohort**				**Unmatched cohort**		
	**Telmisartan group (*n =* 1,503)**	**Non-telmisartan group (*n =* 4,509)**	***p***	**SMD**	**Telmisartan group (*n =* 2,193)**	**Non-telmisartan group (*n =* 17,817)**	**p**
Age, year-old	62.2 ± 13.0	62.4 ± 13.4	0.47	−0.02	62.3 ± 12.6	63.5 ± 13.5	< 0.001
Women, *n* (%)	632 (42.0)	1,905 (42.2)	0.92	0.00	899 (41.0)	7,687 (43.1)	0.058
Current smoking, *n* (%)	120 (8.0)	348 (7.7)	0.78	0.01	128 (5.8)	1,248 (7.0)	0.046
Alcohol, *n* (%)	169 (11.2)	486 (10.8)	0.65	0.01	179 (8.2)	1,504 (8.4)	0.687
SBP, mmHg	134.8 ± 17.1	134.7 ± 16.8	0.77	0.01	134.3 ± 16.6	134.1 ± 16.9	0.636
DBP, mmHg	80.2 ± 13.0	80.7 ± 13.3	0.23	−0.04	80.3 ± 12.5	80.2 ± 13.1	0.501
**Comorbidities**							
Diabetes mellitus, *n* (%)	908 (60.4)	2,747 (60.9)	0.75	−0.01	1,174 (53.5)	9,544 (53.6)	0.995
Dyslipidemia, *n* (%)	1,130 (75.2)	3,420 (75.8)	0.63	−0.02	1,560 (71.1)	12,590 (70.7)	0.664
Chronic kidney disease, *n* (%)	563 (37.5)	1,627 (36.1)	0.35	0.03	576 (26.3)	5,992 (33.6)	< 0.001
Malignancy, *n* (%)	257 (17.1)	734 (16.3)	0.48	0.02	298 (13.6)	2,723 (15.3)	0.039
**Medication**							
Beta-blocker, *n* (%)	362 (24.1)	1,067 (23.7)	0.77	0.01	556 (25.4)	6,755 (37.9)	< 0.001
CCB, *n* (%)	1,283 (85.4)	3,861 (85.6)	0.83	−0.01	1,853 (84.5)	13,756 (77.2)	< 0.001
HCT/Chlorthalidone, n (%)	193 (12.8)	608 (13.5)	0.55	−0.02	277 (12.6)	3,522 (19.8)	< 0.001
Furosemide, *n* (%)	103 (6.9)	333 (7.4)	0.53	−0.02	118 (5.4)	1,911 (10.7)	< 0.001
Other diuretics, *n* (%)	60 (4.0)	190 (4.2)	0.77	−0.01	82 (3.7)	1,463 (8.2)	< 0.001
Aspirin, *n* (%)	374 (24.9)	1,120 (24.8)	>0.99	0.00	543 (24.8)	5,156 (28.9)	< 0.001
Other antiplatelet, *n* (%)	245 (16.3)	758 (16.8)	0.68	−0.01	342 (15.6)	2,968 (16.7)	0.217
Anticoagulant, *n* (%)	97 (6.5)	289 (6.4)	>0.99	0.00	160 (7.3)	1,258 (7.1)	0.718
Statin, *n* (%)	881 (58.6)	2,668 (59.2)	0.73	−0.01	1,254 (57.2)	10,076 (56.6)	0.59
Insulin, *n* (%)	82 (5.5)	246 (5.5)	>0.99	0.00	83 (3.8)	974 (5.5)	0.001
Platinum-based chemotherapeutics, *n* (%)	69 (4.6)	199 (4.4)	0.83	0.01	69 (3.1)	458 (2.6)	0.129
Aminoglycosides, *n* (%)	19 (1.3)	51 (1.1)	0.78	0.01	20 (0.9)	223 (1.3)	0.205
Acetaminophen, *n* (%)	322 (21.4)	944 (20.9)	0.72	0.01	363 (16.6)	3,341 (18.8)	0.013
NSAIDs, *n* (%)	292 (19.4)	877 (19.4)	>0.99	0.00	345 (15.7)	2,791 (15.7)	0.96
Antimalarial drugs, *n* (%)	8 (0.5)	31 (0.7)	0.64	−0.02	8 (0.4)	144 (0.8)	0.033
PDE5 inhibitors, *n* (%)	9 (0.6)	28 (0.6)	>0.99	0.00	18 (0.8)	173 (1.0)	0.571
Other ototoxic, *n* (%)	17 (1.1)	59 (1.3)	0.69	−0.02	26 (1.2)	317 (1.8)	0.043
Ototoxic drug ≥ 1	776 (51.6)	2,304 (51.1)	0.74	0.01	1,014 (46.2)	9,312 (52.3)	< 0.001
Ototoxic drug ≥ 2	295 (19.6)	894 (19.8)	0.90	−0.01	338 (15.4)	3,405 (19.1)	< 0.001
Ototoxic drug ≥ 3	101 (6.7%)	311 (6.9)	0.86	−0.01	107 (4.9)	1,173 (6.6)	0.002
**Laboratory results**							
Total cholesterol, mg/dL	164.2 ± 43.1	166.1 ± 42.4	0.15	−0.05	164.3 ± 42.8	165.2 ± 42.3	0.44
Triglyceride, mg/dL	155.1 ± 157.1	156.8 ± 151.4	0.74	−0.01	155.8 ± 156.2	157.8 ± 130.8	0.674
Glucose, mg/dL	126.9 ± 48.5	125.5 ± 47.0	0.34	0.03	126.8 ± 47.5	126.4 ± 47.1	0.742
High sensitivity CRP, mg/L	8.8 ± 32.2	7.8 ± 24.4	0.40	0.04	8.7 ± 32.0	9.5 ± 30.0	0.503

CCB, calcium channel blocker; DBP, diastolic blood pressure; CRP, C reactive protein; HCT, Hydrochlorothiazide; NSAID, nonsteroidal anti-inflammatory drug; PDE5, Phosphodiesterase-5; SBP, systolic blood pressure; SMD, standardized mean difference.

### 3.2 Incident of hearing loss

**Figure 1:**
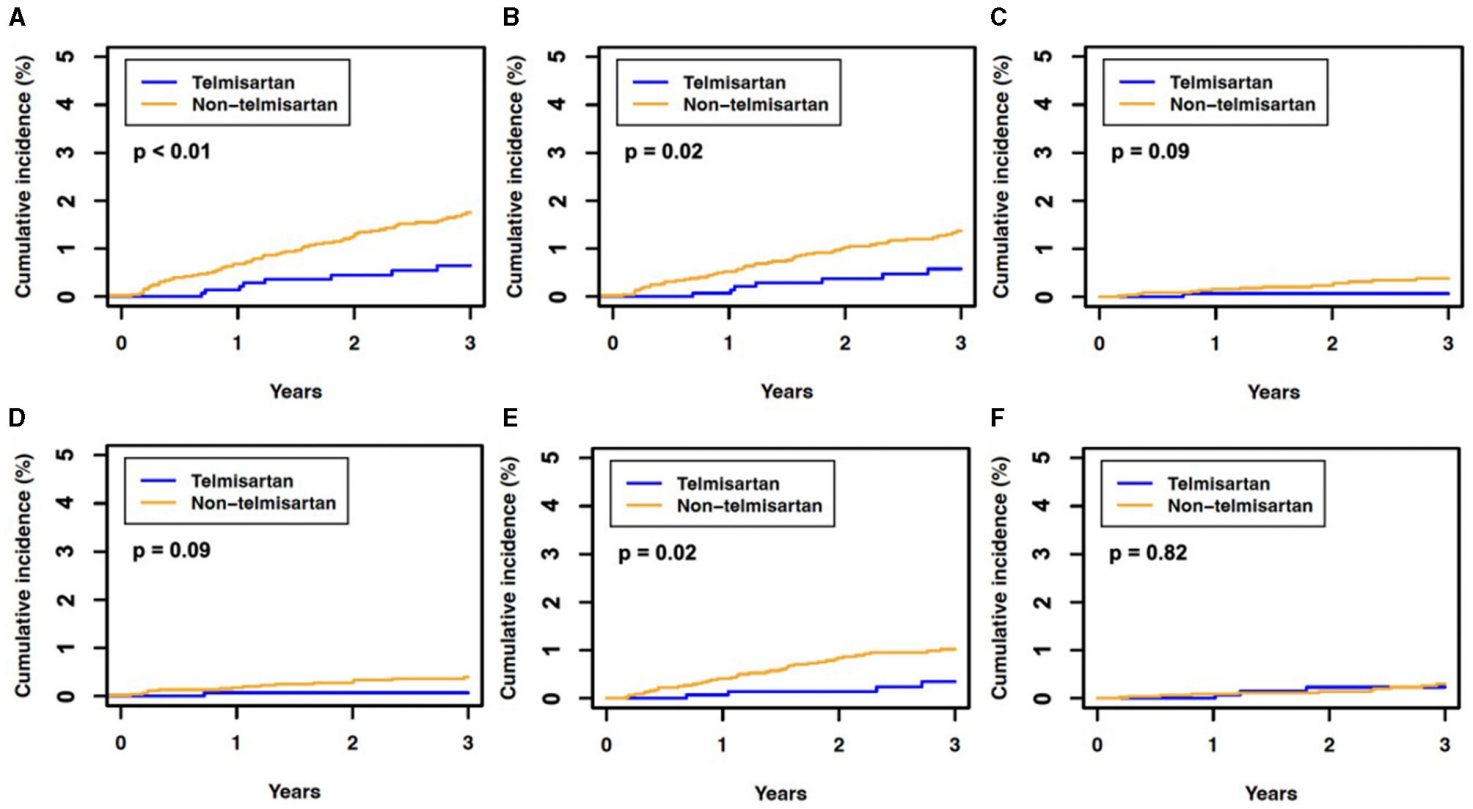
The 3-year cumulative incidence of hearing loss in the propensity score-matched cohort. **(A)** Total hearing loss, **(B)** bilateral hearing loss, **(C)** unilateral hearing loss, **(D)** mild hearing loss, **(E)** moderate hearing loss, and **(F)** severe hearing loss or deafness. The *P*-value was determined using Kaplan–Meier estimates and a log-rank test, with *P* < 0.05 indicating statistical significance.

**Figure 2:**
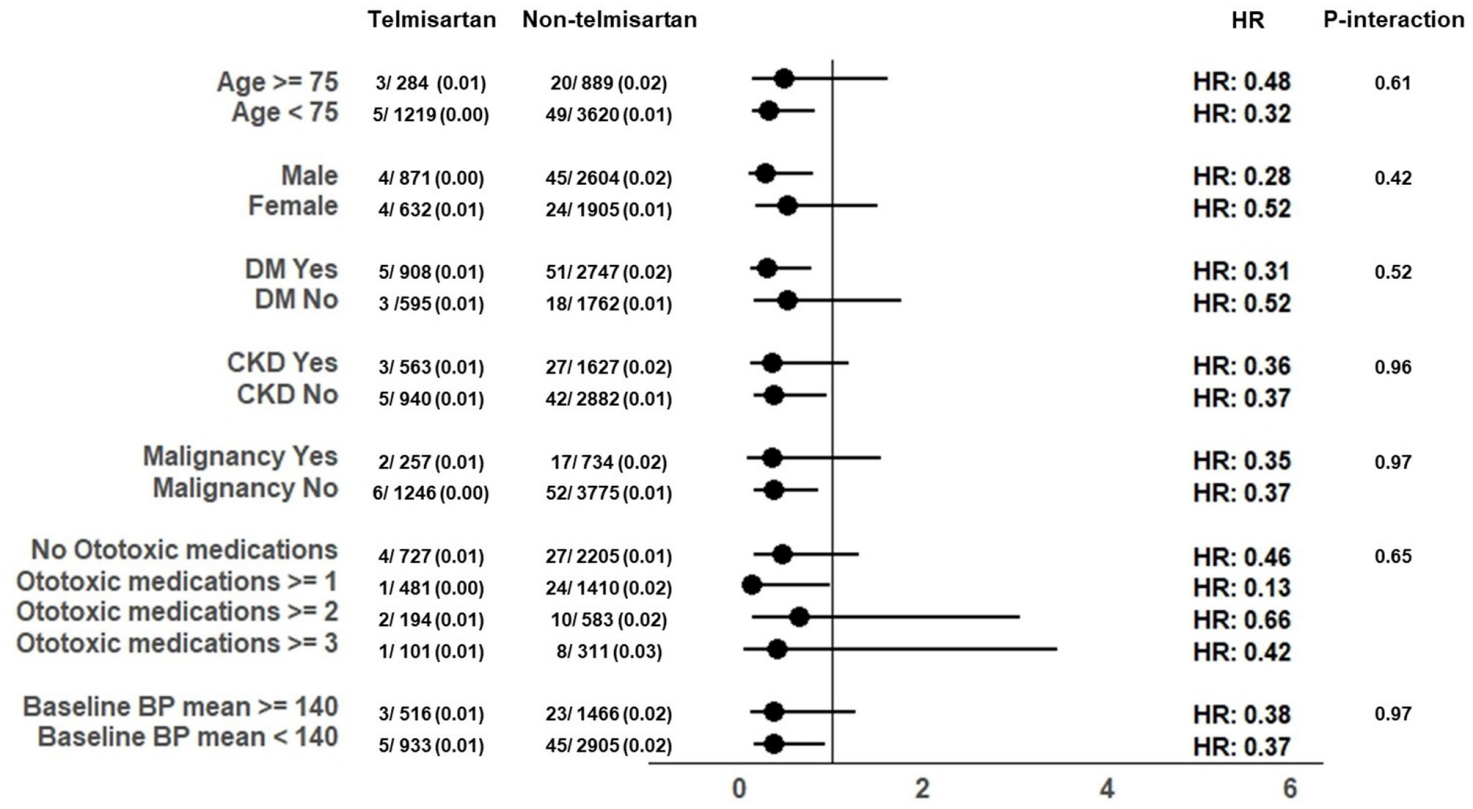
Exploratory subgroup analysis in 3-year hearing loss according to the telmisartan.

The telmisartan and non-telmisartan groups showed significant differences in the incidence of hearing loss (Table 2). A significantly lower incidence of total hearing loss was observed in the telmisartan group compared to the non-telmisartan group (0.5% vs. 1.5%, log-rank *p* = 0.005, Figure 1A). Reduction of hearing loss was predominantly shown in bilateral hearing loss (0.5% vs. 1.2%, log-rank *p* = 0.022, Figure 1B). In contrast, there was a decreased incidence of hearing loss regarding unilateral hearing loss (0.1% vs. 0.3%, log-rank *p* = 0.092, Figure 1C). Both bilateral and unilateral hearing losses in our study were categorized as sensorineural hearing loss. However, only one case in the non-telmisartan group showed conductive hearing loss, which presented as bilateral hearing loss. Regarding the severity, the telmisartan group had a numerically lower incidence of mild hearing loss than the non-telmisartan group, without statistical significance (0.1% vs. 0.4%, log-rank *p* = 0.091, Figure 1D). However, moderate hearing loss was significantly less common in the telmisartan group than in the non-telmisartan group (0.3% vs. 0.9%, log-rank *p* = 0.016, Figure 1E). Meanwhile, there was no difference in the incidence of severe deafness between the two groups (Figure 1F). In subgroup analysis, this study showed consistent results that lower incidence of total hearing loss was higher in the telmisartan group than in the non-telmisartan group (Figure 2). In addition, there were no differences in the incidences of negative control outcomes between the two groups (Supplementary Table 1).

**Table 2:** Three-year incidence of hearing loss between the telmisartan and non-telmisartan group in propensity score matched cohort.

	**Telmisartan group (*n =* 1,503)**	**Non-telmisartan group (*n =* 4,509)**	**Telmisartan vs Non-telmisartan HR (95% CI)**	**p-value (log-rank test)**
Total hearing loss	8 (0.5)	69 (1.5)	0.37 (0.18–0.76)	0.005
Bilateral hearing loss	7 (0.5)	54 (1.2)	0.41 (0.19–0.91)	0.022
Unilateral hearing loss	1 (0.1)	15 (0.3)	0.21 (0.03–1.57)	0.092
**Severity**				
Mild	1 (0.1)	16 (0.4)	0.21 (0.03–1.56)	0.091
Moderate	4 (0.3)	41 (0.9)	0.30 (0.11–0.85)	0.016
Severe or deafness	3 (0.2)	11 (0.2)	0.86 (0.24–3.08)	0.815

CI, confidence interval; HR, hazard ratio.

## 4 Discussion

The present study reported the association between the angiotensin II receptor blocker telmisartan and the prevention of hearing loss in patients with hypertension. After PSM, the telmisartan group showed less incidence of hearing loss events during the 3-year follow-up compared to the non-telmisartan group. In addition, less incidence of hearing loss in the telmisartan group was consistent with subgroup analysis. This is the first clinical evidence using a large-scale registry that the incidence of hearing loss may differ regarding telmisartan usage.

Telmisartan has demonstrated significant therapeutic benefits for cardiovascular diseases. Key studies, such as ONTARGET and TRANSCEND, have primarily focused on patients with atherosclerotic disease or diabetes with organ damage, revealing a lower incidence of major cardiovascular events in patients with hypertension treated with telmisartan (6, 7). Recently, a study reported that comparing the mid-term cardiovascular effects of telmisartan with other ARBs in patients with hypertension requiring multiple antihypertensive drugs, showing a particularly lower incidence of new-onset dialysis compared to other ARBs, and positioning it as a viable alternative therapy in patients with cardiovascular disease or high-risk diabetes (11). Telmisartan has been well studied, and it exhibits several beneficial characteristics including enhanced insulin sensitivity (12, 13), anti-inflammatory and antioxidant actions in the kidneys (14, 15), and improved function of the left ventricle of the heart (16, 17) due to its PPAR γ agonistic properties (18). Meanwhile, PPAR γ signaling pathways are believed to be mediated by the protective mechanism of the auditory cells (19). However, there is no data on the association between telmisartan and hearing loss in clinical practice. Our study showed an association between telmisartan and hearing loss prevention in patients with hypertension in real-world practice.

The association between PPAR γ agonists and hearing loss encompasses a range of protective mechanisms against noise-induced cochlear damage and ototoxicity (5, 20, 21). Noise-induced hearing loss has been linked to the down-regulation of PPARs, influenced by the interplay between oxidative stress and inflammation. Studies indicate that oxidative stress is a primary factor in cochlear injury due to noise, with increased inflammation resulting from PPAR down-regulation caused by reactive oxygen species (ROS) (21). Reducing oxidative stress leads to restoring PPARs to normal levels, thereby re-establishing control over inflammation. PPAR γ agonists, such as thiazolidinedione, target PPAR γ receptors, key transcription factors in glucose and lipid metabolism, inflammation, and organ protection (9). These drugs have been shown to protect auditory hair cells (HCs) from oxidative stress and apoptosis (8). Specifically, pioglitazone has demonstrated efficacy in mitigating gentamicin-induced oxidative stress and apoptosis in mouse organ of Corti explants, countering the rise in ROS and inhibiting the activation of pro-apoptotic mediators. This effect is achieved by regulating genes that control ROS detoxification, suggesting a therapeutic potential for PPAR γ agonists in treating hearing loss (8, 9, 22).

However, clinical trials have yielded mixed results. A phase II trial involving a thermosensitive gel, STR-001, delivered via intratympanic injection to cochlear implant candidates found that pioglitazone was well-tolerated but did not significantly preserve hearing. In a subsequent phase III trial assessing the safety and tolerability of STR-001, both as an intratympanic injection and oral tablets, treatment was well-tolerated in patients with sudden sensorineural hearing loss. However, the primary and secondary endpoints for hearing improvement and speech recognition were inconclusive. Notably, one treatment group observed an increase in non-serious adverse events, such as dizziness and tinnitus (19).

Regarding ototoxicity, a study investigating the effects of telmisartan on ototoxicity provides evidence of its protective role against hearing loss (10, 23). Telmisartan exhibits partial agonism on the PPAR γ. This study focused on the *in vitro* effects of telmisartan on cochlear explants exposed to gentamicin, a known ototoxic agent. The results showed telmisartan-protected auditory HCs against gentamicin-induced ototoxicity. This protective effect was attributed to the PPAR γ signaling pathway, as demonstrated by the complete blocking of the protective effect by GW9662, an irreversible PPAR γ antagonist. Neither exposure to GW9662 nor telmisartan alone was found to be toxic to auditory HCs. Based on these findings, the study concluded that through PPAR γ signaling, telmisartan can potentially protect auditory HCs from gentamicin-induced ototoxicity (23). In context, telmisartan could be considered for future use in preventing or treating sensorineural hearing loss. However, there is no clinical data to show better clinical outcomes regarding preventing hearing loss. The present study showed the first observation of preventing hearing loss in patients using telmisartan in real-world, large-scale patient data.

### 4.1 Limitations

The present study has several limitations. Firstly, we acknowledge the potential for unintentional selection bias in the sampling procedure, as this study relies on a retrospective analysis of the EHR database. To mitigate this bias, PSM and negative control outcome analysis were employed. Nevertheless, additional unquantified variables, such as socio-economic status, which were not accounted for in the EHR database, could influence the results. Furthermore, the occurrence of case-cross-over in both groups could have impacted the findings of this investigation. This phenomenon tends to align the effect estimates with the null hypothesis, perhaps resulting in underestimating the potential advantages of telmisartan treatment. Furthermore, due to the intricate nature of the data, we did not consider the dosage or specific varieties of combined antihypertensive medications, particularly regarding fixed-dose combinations. Before PSM, there are variations in the utilization rates of DHP-CCBs, beta-blockers, and diuretics between the telmisartan and non-telmisartan groups. Nevertheless, the distribution of secondary medications (beta-blockers, DHP-CCB, and diuretics) was evenly balanced between groups following a comprehensive PSM process. Finally, it is important to note that while these factors suggest a possible association between telmisartan and hearing loss, the relationship is complex and not fully understood. More research is needed to clarify the extent of this association, understand the underlying mechanisms, and determine whether using telmisartan can help prevent or mitigate hearing loss.

## Data Availability

The original contributions presented in the study are included in the article/Supplementary material, further inquiries can be directed to the corresponding author.
