# Supplementary material for: Association of the protective effect of telmisartan on hearing loss among patients with hypertension
Source: Front Neurol. 2024 Aug 27;15:1410389. doi: 10.3389/fneur.2024.1410389 (PMC11384575; doi:10.3389/fneur.2024.1410389)
Supplement: Supplementary file 1 [file Table_1.docx]

**Supplemental table 1**. Negative control outcomes in propensity score matched cohort

|  | **Telmisartan group**  **(n = 1,503)** | **Non-telmisartan group**  **(n = 4,509)** | **p** |
| --- | --- | --- | --- |
| Foot fracture | 2 (0.1) | 8 (0.2) | 0.767 |
| Leg fracture | 1 (0.1) | 8 (0.2) | 0.374 |
| Hip fracture | 3 (0.2) | 22 (0.5) | 0.161 |
| Forearm fracture | 1 (0.1) | 10 (0.2) | 0.265 |
| Hand fracture | 1 (0.1) | 6 (0.1) | 0.544 |
| Traumatic subdural hemorrhage | 1 (0.1) | 9 (0.2) | 0.323 |
| Peptic ulcer | 16 (1.1) | 59 (1.3) | 0.545 |
| Pneumonia | 22 (1.5) | 81 (1.8) | 0.477 |
| Urinary tract infection/cystitis | 23 (1.5) | 62 (1.4) | 0.573 |
| Conjunctivitis | 3 (0.2) | 15 (0.3) | 0.464 |
| Herpes zoster | 12 (0.8) | 21 (0.5) | 0.100 |
| Contact dermatitis | 1 (0.1) | 8 (0.2) | 0.377 |
| Urticaria | 9 (0.6) | 21 (0.5) | 0.456 |
| Psoriasis | 4 (0.3) | 11 (0.2) | 0.837 |
| Cataract | 51 (3.4) | 164 (3.6) | 0.827 |
| Glaucoma | 36 (2.4) | 127 (2.8) | 0.513 |
| Lung cancer | 6 (0.4) | 17 (0.4) | 0.830 |
| Gastric cancer | 1 (0.1) | 18 (0.4) | 0.088 |
| Liver cancer | 4 (0.3) | 13 (0.3) | 0.936 |
| Colorectal cancer | 5 (0.3) | 17 (0.4) | 0.853 |
| Prostate cancer | 2 (0.1) | 14 (0.3) | 0.279 |
| Breast cancer | 5 (0.3) | 9 (0.2) | 0.325 |
| Rheumatoid arthritis | 11 (0.7) | 27 (0.6) | 0.524 |
| Osteoporosis | 30 (2.0) | 64 (1.4) | 0.084 |
| Stroke | 3 (0.2) | 16 (0.4) | 0.386 |
| Myocardial infarction | 18 (1.2) | 43 (1.0) | 0.354 |
| Death | 42 (2.8) | 130 (2.9) | 0.991 |
